# Evaluation of Three Automated Genome Annotations for *Halorhabdus utahensis*

**DOI:** 10.1371/journal.pone.0006291

**Published:** 2009-07-20

**Authors:** Peter Bakke, Nick Carney, Will DeLoache, Mary Gearing, Kjeld Ingvorsen, Matt Lotz, Jay McNair, Pallavi Penumetcha, Samantha Simpson, Laura Voss, Max Win, Laurie J. Heyer, A. Malcolm Campbell

**Affiliations:** 1 Department of Biology, Davidson College, Davidson, North Carolina, United States of America; 2 Microbiology, Department of Biological Sciences, Aarhus University, Aarhus, Denmark; 3 Department of Mathematics, Davidson College, Davidson, North Carolina, United States of America; University of Maryland, United States of America

## Abstract

Genome annotations are accumulating rapidly and depend heavily on automated annotation systems. Many genome centers offer annotation systems but no one has compared their output in a systematic way to determine accuracy and inherent errors. Errors in the annotations are routinely deposited in databases such as NCBI and used to validate subsequent annotation errors. We submitted the genome sequence of halophilic archaeon *Halorhabdus utahensis* to be analyzed by three genome annotation services. We have examined the output from each service in a variety of ways in order to compare the methodology and effectiveness of the annotations, as well as to explore the genes, pathways, and physiology of the previously unannotated genome. The annotation services differ considerably in gene calls, features, and ease of use. We had to manually identify the origin of replication and the species-specific consensus ribosome-binding site. Additionally, we conducted laboratory experiments to test *H. utahensis* growth and enzyme activity. Current annotation practices need to improve in order to more accurately reflect a genome's biological potential. We make specific recommendations that could improve the quality of microbial annotation projects.

## Introduction

The field of genomics has become increasingly important in the world of science. The ability to collect and analyze genomic data provides great potential for the study of life, and is especially useful with multiple organisms living in one community and with organisms that cannot easily be grown in culture [1]. Cost-effective sequencing methods and tools have surpassed manual annotation as the amount of input data has increased by orders of magnitude. Modern sequencing methods have given researchers the ability to sequence up to one gigabase in a single run [2]. In order to benefit from the power of genomic sequencing, the annotation tools must be reliable and the databases must be consistent. In the coming years, hundreds of genomes will be submitted to be sequenced and annotated [3]. Consequently, automated annotation needs to be as accurate as possible. Every time a particular annotation service repeats a systematic error, the results are deposited into a database. Wet-lab experiments rarely accompany annotations in large part due to the scale of the problem. However, as new annotations are produced by the same service, previously deposited errors are used to validate the newest annotation, which contains the same systematic errors. As a result, systematic errors are used to validate repetition of the same errors, and the databases accumulate incorrect annotations that are particular to each annotation service.

The goal of this study was to compare three annotation services: The Joint Genome Institute's (JGI) Integrated Microbial Genome (IMG) system [4], the National Microbial Pathogen Data Resource's (NMPDR) Rapid Annotation using Subsystems Technology (RAST) server [5], and the J. Craig Venter Institute (JCVI) Annotation Service (http://www.jcvi.org/cms/research/projects/annotation-service/). A secondary goal was to examine the *Halorhabdus utahensis* genome in order to understand the physiology and metabolic potential of a previously un-annotated genome. The halophilic archaeon *H. utahensis* was isolated from the Great Salt Lake, Utah. *H. utahensis* grows optimally in 27% NaCl at 50°C [6]. We compared the annotations of three automated services and documented distinct differences in annotation output. We located the origin of replication and the consensus ribosome-binding sequence for this organism manually because none of the services attempted to locate them. We incubated the organism with several carbon substrates and tested for growth and enzyme activity based on the automated annotations, and we were not able to detect some of the predicted enzymes. Based on our comparison, we developed a series of recommendations to improve the annotation services and ultimately the quality of DNA sequence databases.

## Results

The *H. utahensis* genome is comprised of 3,129,561 base pairs that encode approximately 3,000 genes, depending on the annotation service. In our analysis, we compared IMG, RAST, and JCVI annotations by examining gene calls, gene counts, start/stop sites, Enzyme Commission (EC) numbers, and pathways. We also examined evidence to determine the consensus ribosome-binding sequence and the DNA replication initiation site. To establish uniform annotation procedures, we manually annotated the RNA genes first because they provided a manageable number of genes to complete and are highly conserved across species [7].

### Comparison of rRNA calls

We first examined the gene calls for ribosomal RNA. IMG and RAST called three rRNA genes, whereas JCVI called only two (Table 1). IMG and RAST called identical 5s, 16s, and 23s rRNA genes, but JCVI called only the 5s and 16s rRNA, not the 23s rRNA gene. JCVI's 5s rRNA differed in start site from the other two annotations by a single base pair. JCVI's 16s rRNA differed in start site by 18 base pairs and stop site by 986 base pairs.

**Table 1:** Comparison of rRNA calls.

IMG	DNA coordinates	Length
16s rRNA	2397347..2398825 (+)	1479 bp
23s rRNA	2399190..2402100 (+)	2911 bp
5s rRNA	2402216..2402338 (+)	123 bp
**RAST**		
16s rRNA	2397347.. 2398825 (+)	1479 bp
23s rRNA	2399190.. 2402100 (+)	2911 bp
5s rRNA	2402216.. 2402338 (+)	123 bp
**JCVI**		
16s rRNA	2397365.. 2397839 (+)	475 bp
5s rRNA	2402217.. 2402338 (+)	122 bp

Review of predicted coding regions for ribosomal RNA for each annotation service shows that IMG and RAST have identical calls, while JCVI fails to call 23s rRNA and predicts different start and stop sites.

### Consensus RBS

Ribosomes are often recruited for translation by a sequence closely upstream of the coding region [8]. These short, purine-rich sequences are specific to individual species. Because ribosome-binding sites (RBS) often reside several base pairs upstream of the start codon, finding a species-specific consensus RBS sequence could prove useful in determining start sites. None of the three annotation services attempted to find a species-specific RBS.

**Figure 1:**
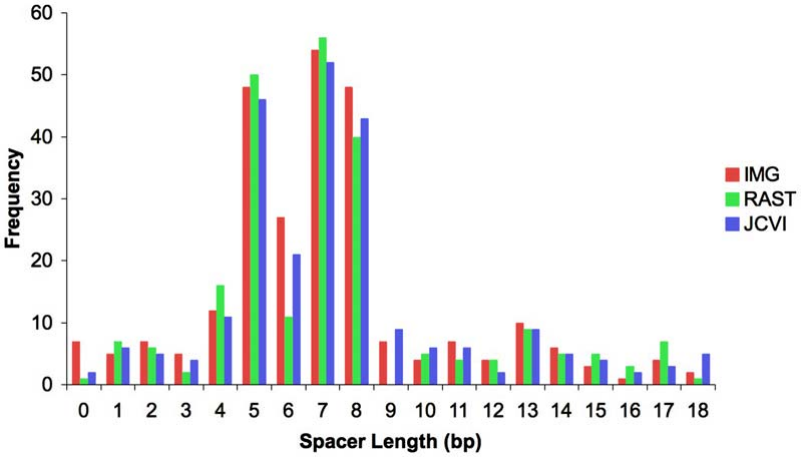
Comparison of RBS spacer lengths. Histogram displaying the frequency of the consensus RBS at varying spacer lengths. Spacer length refers to the number of base pairs between the 3′ end of the RBS and the 5′ end of the start codon for each gene. The consensus RBS generally lies between 4 and 8 base pairs upstream of the start codon.

In order to find the consensus RBS, we analyzed the regions upstream of all predicted genes for each of the three annotations. We used RSAT's Pattern Discovery Tool to search for prevalent sequence segments in the 50 bases upstream of each predicted gene. We found that the most common 7-base pattern in each annotation was GGAGGTG. This sequence matched the complementary sequence (CACCUCC) at the 3′ end of the 16s rRNA, the Shine-Dalgarno sequence, and validated GGAGGTG as the RBS [9]. We observed that in *H. utahensis*, the RBS most often lies between four and eight base pairs upstream of the start codon (Figure 1). Interestingly, the consensus RBS was six bases upstream of the start codon less often than either five or seven bases. We knew that RBS sites do not always match perfectly to the consensus sequence, so we searched the upstream regions of called genes for sequences with zero or one base differing from GGAGGTG. For instance, the search would identify GGCGGTG as a valid RBS, but would not select GGCGGT*A* . We found that under these criteria, a match to the RBS was present upstream of fewer than 10 percent of the predicted genes in each of the three annotations (IMG: 8.6%, RAST: 8.1%, JCVI: 8.6%). Next, we allowed the search to include sequences with up to a two bases different from the consensus RBS in order to find the prevalence of a more degenerate RBS. With the less stringent criteria, we determined the ratio of upstream sequences with a putative RBS to be just below 50 percent (IMG: 48.2%, RAST: 47.7%, JCVI: 48.7%). Therefore, annotation services should search for RBS sites with at least 2 degenerate bases to maximize their ability to more definitively determine the start codons.

### Intron-containing tRNA genes

In reviewing the tRNA gene calls made by each annotation service, we found that IMG and RAST called the same 45 tRNAs, while JCVI called 44. JCVI had failed to call an intron-containing tRNA-met, which IMG and RAST successfully called (tRNA-met, 1998587..1998721 (+), 135 bp). Because ATG is the only codon for methionine, this omission by JCVI is significant. Further investigation revealed another oddity— none of the annotation services had called a gene that coded for tRNA-trp, which has TGG as its only codon. Through additional searches, we found that the *H. utahensis* genome contains a gene coding for a tRNA intron endonuclease similar to that of *Halobacterium volcanii*, another halophilic archaeon [10]. We obtained the tRNA-trp sequence from *H. volcanii* from the Genomic tRNA Database, and BLASTed the sequence against the *H. utahensis* genome (http://lowelab.ucsc.edu/GtRNAdb/). The search revealed a tRNA-trp in the *H. utahensis* genome with 90 percent identity, containing a 103-base intron (tRNA-trp, 465601..465777 (−), 177 bp). Therefore, all three annotation services failed to identify the tRNA-trp gene.

### Origin of replication

**Figure 2:**
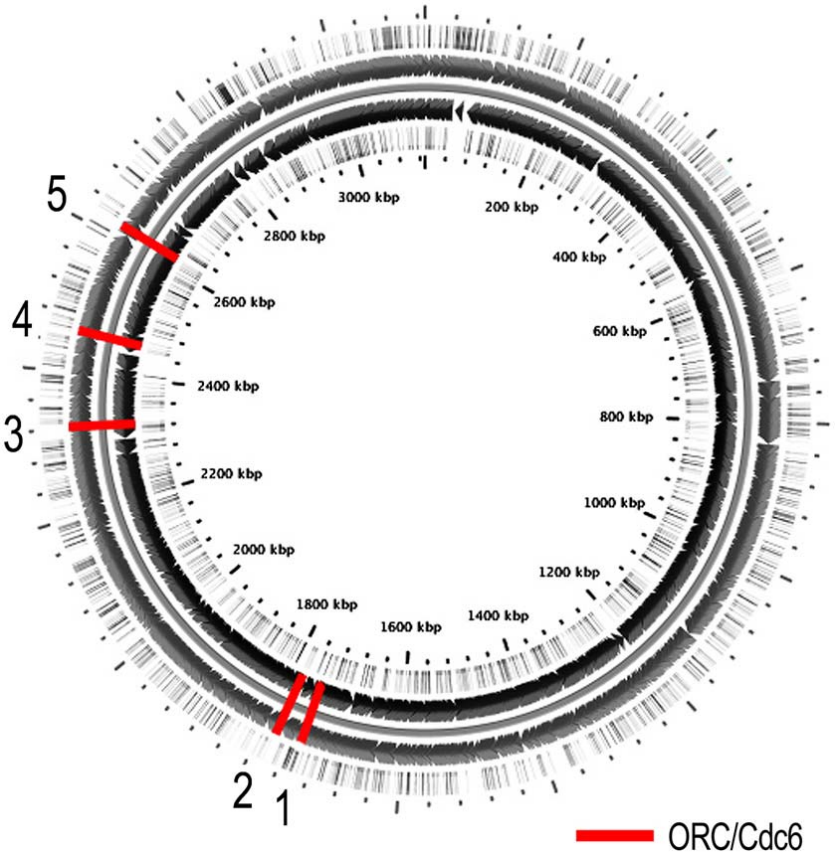
*H. utahensis* primary contig and ORC/Cdc6 genes. Circular display of the largest contig of the *H. utahensis* genome sequence. The contig begins at the top and wraps clockwise. The red bars illustrate the location of ORC/Cdc6 orthologs. The ORC/Cdc6 gene numbered 3 lies near the origin of replication, at 2,327,225 base pairs.

Replication initiation has been studied extensively in bacteria and archaea, all the three annotation services confirmed via email that they do not attempt to locate the DNA replication site in either bacterial or archaeal genomes. We located the DNA replication initiation site by searching for evidence outlined in several papers concerning archaeal origins of replication [11], [12]. First, we located genes in the *H. utahensis* genome that code for the archaeal equivalent of an Origin Recognition Complex subunit (ORC) and a cell division control protein (Cdc6). These ORC/Cdc6 genes are good indicators of the initiation site because they are often located in close proximity to the replication origin. We identified five ORC/Cdc6 orthologs in *H. utahensis*. Due to the proximity of DNA polymerase, helicase, and other replication factor genes, we examined the area surrounding ORC/Cdc6 gene 3 (2324949..2326724 (−)) (Figure 2).

Upon closer investigation, we found supporting evidence that this region contains the origin of replication. We discovered a non-coding, AT-rich, 1,000 base pair region (2326724..2327725) upstream of ORC/Cdc6 gene 3. This AT-rich region was 49 percent GC which is substantially different from the 63 percent genome-wide average. This region also contained a pair of 28-base inverted repeats, which form a transcription factor binding site when coiled (2327117..2327142 (+), 2327719..2327745 (−)) [13]. Other supporting evidence includes opposite-facing genes and a local minimum in cumulative GC skew [14]. Therefore, we hypothesize that the origin of replication for *H. utahensis* is located at base 2,327,225 of the primary contig.

### Comparison of gene calls

**Figure 3:**
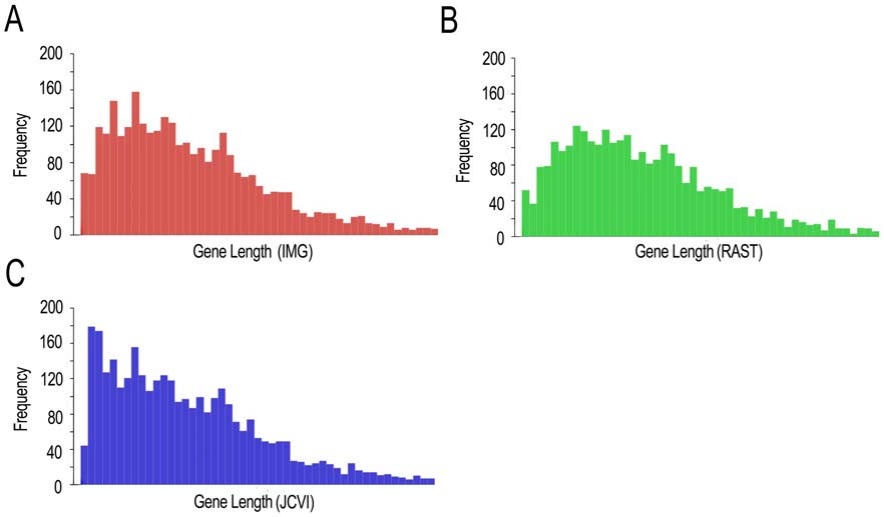
Comparison of gene length frequencies. Histograms displaying gene length illustrate similarities and differences between annotations. (A) Frequency of genes called by IMG at different lengths. (B) Frequency of genes called by RAST at different lengths. (C) Frequency of genes called by JCVI at different lengths. JCVI has a higher frequency of short genes called than IMG or RAST.

Gene predictions varied considerably between annotation services due to differences in annotation methods and criteria for gene calls. The number of predicted genes ranged from 2,898 to 3,254, and the average gene length ranged between 845 and 942 base pairs (Table 2). JCVI predicted the largest number of genes, followed by IMG, then RAST. However, RAST called considerably longer genes on average than IMG or JCVI (Figure 3). Our comparison illustrates the variation between the different annotation services working with the same genomic sequence.

**Table 2:** Comparison of descriptive statistics.

Annotation	Genes	Mean	Median	Minimum	Maximum
**IMG**	3097	869.9 bp	728 bp	70 bp	7130 bp
**RAST**	2898	941.8 bp	801.5 bp	70 bp	100001 bp
**JCVI**	3254	844.9 bp	692 bp	73 bp	100001 bp

Mean, median, minimum, and maximum gene lengths of the total predicted coding regions illustrate differences in gene calls between the annotations.

**Figure 4:**
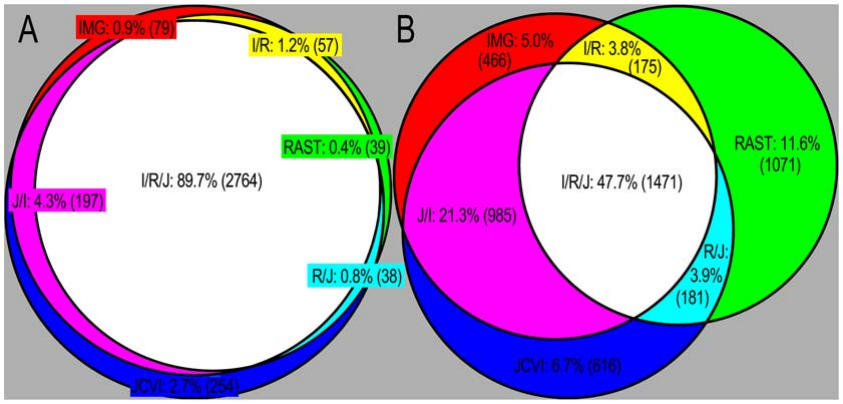
Venn diagrams of gene predictions. (A) The diagram to the left shows the number of predicted protein coding genes that share stop sites with the other annotations. Overlapping regions indicate genes having same stop site between annotations. (B) The diagram to the right shows the number of predicted protein coding genes that share start site and stop site with the other annotations. Overlapping regions indicate genes having exact matches between annotations.

A single stretch of DNA can be annotated in a number of different ways. Discrepancies between gene calls in the same region can be categorized as differences in start site or differences in reading frame. The annotations agreed on reading frames much more often than they agreed on start site (Figure 4). JCVI had the largest number of genes with unique reading frame. However, 89.7 percent of all predicted protein coding genes shared the same stop sites in all three annotations. When comparing exact matches, IMG and JCVI shared more exact-match gene calls with one another than with RAST. RAST had the largest number of start codon calls that differed from the other two annotations. Only 47.7 percent of the predicted protein-coding regions were identical in all three annotations.

**Figure 5:**
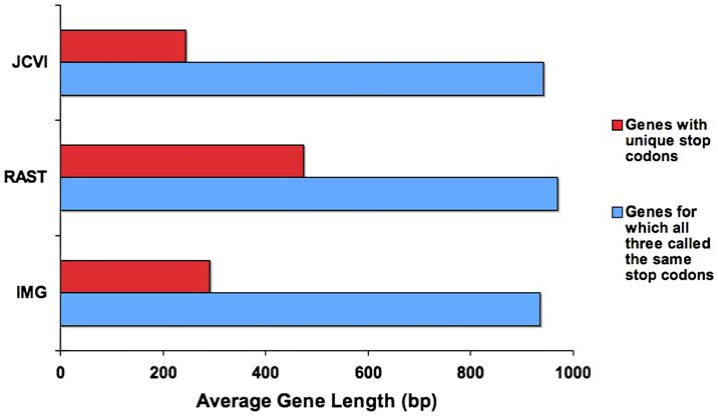
Comparison of average gene length. Illustrates average gene length for two categories. Red bars represent the average length of genes from each annotation that have distinct stop codons. Blue bars represent the average length of genes that have a common stop codon across the three annotations.

Of the genes with identical reading frames in all three annotations, those called by RAST had the longest average length; 967 base pairs for RAST, 940 for JCVI, and 934 for IMG. For genes unique to one annotation, RAST calls had the longest average length, followed by IMG, then JCVI (Figure 5).

To further analyze differences in start site annotations, we tabulated the start codon for each predicted gene. ATG was the most common start codon across all annotations, accounting for 75.7 percent of the starts. RAST contained the largest proportion of alternative start codons, with 39.0 percent of the genes predicted to begin with a codon other than ATG. IMG and JCVI had considerably lower alternative start codon usage, with 19.9 and 14.3 percent, respectively (Table 3).

**Table 3:** Comparison of start codons.

Annotation	Genes	ATG start	Other start	% Not ATG
**IMG**	3047	2604	443	14.3%
**RAST**	2851	1723	1128	39.0%
**JCVI**	3208	2562	646	19.9%

Analysis of predicted protein coding genes displays incidence of ATG and alternative start codons for each annotation. RAST has a greater tendency to call genes with alternative start codons than the other annotation services.

A gene that exemplifies the differences in start codon selection between annotation services is a putative glycoside hydrolase (Table 4). While each annotation predicted the same stop codon for the gene, they all differed in the selection of a start codon. RAST predicted GTG as the start codon, whereas IMG and JCVI identified ATGs at two different locations. The difference in start codon also caused the gene length to differ for each annotation. Without knowing the species-specific RBS, the annotation services used different criteria to call start codons. These annotations must be incorrect two out of three times if the three annotations disagree with each other.

**Table 4:** Comparison of putative glycoside hydrolase start sites.

Annotation	DNA coordinates	Start codon	Length
**IMG**	69942..72866 (+)	ATG	2925 bp
**RAST**	69912..72866 (+)	GTG	2955 bp
**JCVI**	69882..72866 (+)	ATG	2985 bp

Examination of an individual gene displays tendencies of the annotation services. RAST identifies GTG as the start codon for the gene, while IMG and JCVI select two ATG codons at different locations. Predicted start codon affects gene length.

### Genes and pathways

The addition of EC numbers to predicted genes provides a specific, universal classification for enzymes [15]. EC numbers facilitate a common language for enzymes in pathways and subsystems. We tallied the predicted genes in each annotation that had been labeled with either full or partial EC numbers. We found that RAST assigned 597 (20.9 percent) of its genes with an EC number. JCVI assigned EC numbers to 485 (15.1 percent) of its genes while IMG assigned only 294, or 9.6 percent of its genes with an EC number.

Like EC numbers, gene names serve as indicators of the protein's family and function. Each of the annotation services attempted to provide protein names for their calls. Unlike EC numbers, there is no standard for gene names, which can lead to annotation problems. The naming discrepancies make similarities between annotations unclear. For example, a predicted gene ending at base 807,321 was called by all three annotations. Although the annotations agreed on the protein's amino acid sequence, they all named it differently (Table 5). IMG called it a “cation diffusion facilitator family transporter,” RAST called it a “cobalt-zinc-cadmium resistance protein,” and JCVI called it a “cation efflux protein.” It is possible to recognize the relatedness of the terms, but this lack of standardization does not facilitate high-throughput comparisons and drives up the percentage of the genome annotation workload that must be performed manually by experts.

**Table 5:** Comparison of gene names.

Annotation	DNA coordinates	Gene product name
**IMG**	806410..807321 (+)	Cation diffusion facilitator family transporter
**RAST**	806374..807321 (+)	Cobalt-zinc-cadmium resistance protein
**JCVI**	806374..807321 (+)	Cation efflux protein

The annotation services use different names to identify the same protein.

The example above can be sorted out by a human reading the slightly different annotations, but additional examples of differences in annotation are more difficult to reconcile. For example, locus EEJ07885 submitted to NCBI by JGI was called a peroxiredoxin (EC 1.11.1.15) by both JGI and JVCI, but RAST called the exact same ORF a monooxygenase (EC 1.14.13.-). We wondered if the differences in gene annotation were caused by RAST assigning seven additional amino acids on the amino terminus based on a predicted an alternative start codon. When we submitted the RAST and JGI amino acid sequences to BLASTp, the top hits for both query sequences were monooxygenases, consistent with the RAST annotation. However, the next two highest hits were both peroxiredoxins and consistent with the annotations by JGI and JVCI. One of the peroxiredoxins hits was from the closely related *Halobacterium salinarum* whose genome was annotated by NCBI. When JGI and RAST amino acid sequences were submitted to the conserved domain database (CDD), they both returned the thioredoxin superfamily which includes peroxiredoxins. The example of locus EEJ07885 shows that both the gene name and its associated EC number can be very different depending on which annotation service submits to NCBI. Because NCBI BLAST hits are a critical source of information for all three annotation services, as well as hand-curation by individuals, incorrect annotation can be propagated easily once the annotation is part of NCBI. To clarify this one example of confusing annotation will require careful biochemical analysis of cloned ORFs from the various species to determine if the different annotations reflect biological function or error propagation in the database.

**Figure 6:**
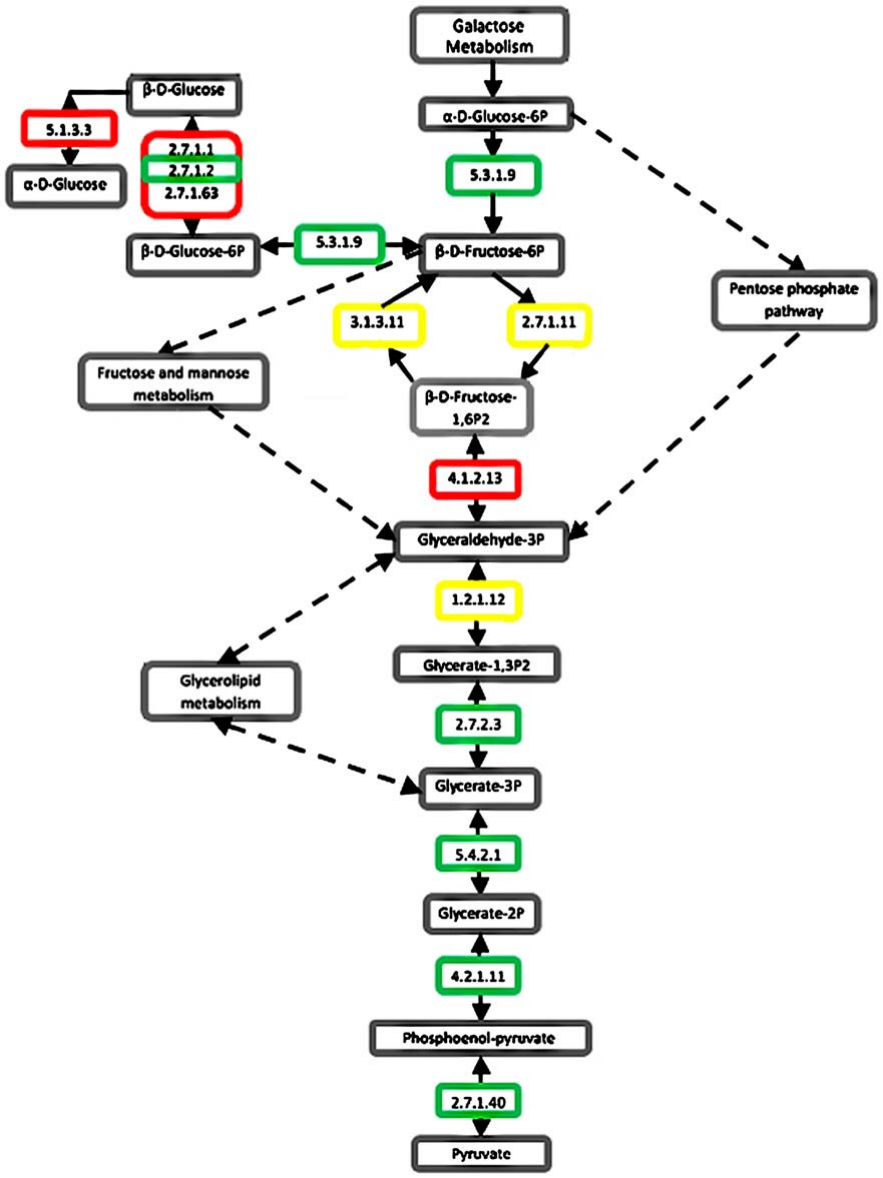
Glycolysis/gluconeogenesis pathway. Diagram based on RAST's KEGG pathway map displays present and absent enzymes for *H. utahensis*. Green boxes indicate that the enzyme was predicted by RAST and displayed in the KEGG map. Yellow boxes designate enzymes that were called by RAST but had not been added to the KEGG map. Red boxes mark enzymes that were listed as absent and could not be located in the *H. utahensis* genome.

Categorizing genes into families and functions enables the construction of genetic pathways. We investigated a number of pathways in the *H. utahensis* genome by using RAST's KEGG analysis feature, which combines the KEGG pathway visualizer with RAST annotation data. We often found RAST's KEGG maps to be incomplete because the diagrams did not include all of the EC-named enzymes predicted in the RAST annotation. For instance, the KEGG map for the glycolysis/gluconeogenesis pathway showed that 7 of the 14 enzymes were not present in *H. utahensis* (Figure 6). However, manual investigation determined that RAST had called three of the missing enzymes without filling in the KEGG map. We reported this error to the RAST curators.

### Laboratory experiments

Automated annotations are helpful starting places if they are biologically accurate. If one annotation service calls a gene incorrectly, subsequent annotations will use this error to repeat the original error. Therefore, the databases and annotations may predict a gene that does not exist in the organism. We tested this possibility with some simple growth tests and enzyme assays under various conditions. We expected growth on various substrates in part because the annotations called four cellulases (43023..45329; 66795..69809; 69912..72866; 1014994..1016439) and a chitinase (55855..57810). We found that *H. utahensis* grows vigorously on xylan, but does not grow on other substrates including cellulose and chitin, despite the fact that the archaeon lives in water crowded with brine shrimp that have chitin exoskeletons. Enzyme assays detected xylanase activity as well as some cellulase activity (Table 6).

**Table 6:** Summary of wet-lab results.

	0 (Control)	Starch	Xylan	CMC-Na	Avicel	Alpha-cellulose	Chitin
**Chitin Azure (Sigma)**	0	0	0	0	0	0	0
**Colloidal-Chitin-RBV**	0	0	0	0	0	0	0
**AZCL-Galacto-mannan (Carob)**	0	0	+	0	0	+	0
**AZCL-Arabino-xylan**	++	++	+++	++	++	++	++
**AZCL-HE-Cellulose**	0	0	+	0	0	0	0
**Ostazin-BR-HE-Cellulose**	0	0	+	0	0	0	0
**AZCL-Xylan (Birchwood)**	++	++	+++	++	++	++	+
**AZCL-Pullulan**	0	0	+	0	0	0	0
**AZCL-Amylose**	0	0	+	0	0	0	0
***Growth***	0	0	+++	0	0	0	0
***Final pH of culture***	7.8	7.6	6.0	7.8	7.7	N/A	7.6

We incubated *H. utahensis* for seven days in GSL-2 medium with various macromolecular substrates. The topmost row lists the macromolecular substrates used as carbon sources. The first nine entries in the leftmost column indicate the substrates used for enzyme assays. Plus signs denote the level of activity, ranging in order of lowest to highest from 0 to +++.

## Discussion

### rRNA and tRNA

JCVI failed to locate one rRNA and truncated another compared to the other two annotations. The reason for JCVI's annotation errors may have been a difference in tools used to find the rRNAs. JCVI used BLAST and Rfam to locate rRNAs, whereas IMG used an IMG RNA database and RAST used a script by Niels Larsen [16].

JCVI also missed a tRNA-met where the other two annotations found it. This omission is interesting because all three annotation services use the program tRNAscan-SE to locate tRNAs. JCVI might have lowered the default cutoff for tRNA length in tRNAscan-SE, which may have passed over the 135 base pair tRNA-met [17]. In the case of the missing tRNA-trp, none of the annotations called the gene most likely missed because the program overlooks potential tRNAs that contain an intron greater than 80 bases. We recommend that all three annotation services be modified to avoid passing over introns and other split RNA genes.

### Consensus RBS

We obtained strong evidence for a species-specific ribosome-binding site sequence. When allowing for up to one base variation from the consensus RBS, we found this sequence upstream of fewer than 10 percent of the genes. This ratio seems low when compared to data from other organisms [18]. However, when we increased the possible variation to two bases, the RBS prevalence increased to almost 50 percent of upstream sequences. Though much remains unknown about ribosome recruitment and binding in archaea, using a species-specific RBS should enhance the accuracy of start codon identification. We recommend that each annotation service determine the species-specific consensus RBS and identify appropriate variations from the consensus sequence in order to find degenerate ribosome-binding sites.

### Gene lengths, starts, and stops

The patterns that emerge from average length, start site and stop site agreement, and start codon sequence data separate RAST and JCVI. IMG gene calls were based on the self-training GeneMarkS software, and fell in the middle in terms of length number of genes [19]. On average, RAST genes were longer than the others possibly due to the increased calling of alternative start sites by RAST. Additionally, JCVI had more short genes than RAST or IMG. This may have been a result of the JCVI calling many short, hypothetical protein genes. The difference between gene calls of JCVI and RAST was intriguing because of their use of similar annotation tools. Both used the Gene Locator and Interpolated Markov Modeler (GLIMMER) tool for the first pass at genes [20]. The differences may come about in variations in the training set given to GLIMMER before genome analysis. For instance, RAST uses a training set based on genes of close phylogenetic neighbors [21]. Substantial changes also may occur during additional analysis through the use of different tools and databases. When possible, we recommend the use of a phylogenetically precise training set in gene calling. We also recommend the use of species-specific RBS sequence data to aid in selecting the correct start codons.

### Wet-lab experiments

We conducted laboratory experiments to test the ability of *H. utahensis* to grow only on xylan, and detected high xylanase but very little cellulase activity in these cultures (Table 6). Cellulase activity was detected when cells were grown on xylan medium but not when they were inoculated into cellulose medium where the cells were unable to grow. This may have occured due to xylan impurities in the cellulase test substrate, or because the xylanases have a low activity towards cellulose. Regardless, the cellulase activity was too low to allow for growth on cellulose.

Based on genome annotations and pathway mapping, we had expected *H. utahensis* to grow on multiple substrates, using a variety of enzymes to metabolize different carbon sources. It seemed reasonable that the halophile could grow on chitin as its only carbon source. *H. utahensis* lives in water crowded with chitinous brine shrimp exoskeletons, and JCVI called a chitinase gene. Also, the *H. utahensis* genome contained several protein export enzymes, so we had expected enzyme secretion to be possible. There are numerous possibilities for the lack of growth and enzymatic activity. Incorrect annotation, non-functional genes, or non-functional protein export could explain our inability to detect extracellular chitinase activity. Whatever the cause, these results suggest that the gap between a putative gene and a fully functional protein are biologically significant. Alternatively, the growth conditions in the lab could have been insufficient to trigger gene activation, meaning the annotations could be correct.

### Ease and functionality of browsers

Each web-based viewer offered helpful tools and features for research and analysis. The ease of exporting DNA or amino acid sequences for genes made IMG/EDU a valuable resource. IMG also facilitated a text search of 57 annotated archaeal genomes followed by a BLAST of a selected gene against *H. utahensis*. However, the inability to BLAST any DNA or amino acid sequence hindered us from finding the tRNA-trp in the genome. For that, we turned to the SEED-viewer within RAST. The most beneficial SEED feature was the quick and easy BLAST function against *H. utahensis* genome. SEED's ease of sorting and searching the entire predicted gene list was helpful as well. RAST's custom KEGG maps allowed us to view specific pathways, yet the maps were often missing called enzymes. JCVI's Manatee browser had a feature that grouped certain genes together based on function. This greatly aided our search for the origin of replication by compiling many genes involved in the process into one page. However, Manatee was sluggish, contained more manually detected errors, and was not intuitive to use. Manatee was designed for manual annotation but it is the most cumbersome of the three to navigate and visualize the data. We recommend that each annotation service borrow the beneficial features from each other to improve their users' experiences and productivity.

### Additional recommendations

Three annotation services interpreted the genomic sequence data of *H. utahensis* differently. IMG, RAST, and JCVI annotation services found 79, 39, and 254 unique genes, respectively. Each service had multiple unique start sites and gene product calls as well as mistakes. The annotation services provide no estimation for the origin of replication or use a species-specific RBS to increase the quality of the annotation. These discrepancies, errors, and shortcomings in the annotation services are not limited to archaeal annotations. Bacterial and archaeal genomes are passed through comparable annotation pipelines, leading to similar results between the two domains.

Incomplete or incorrect annotation occurs in today's annotation services with both bacterial and archaeal genomes as we documented with locus EEJ07885. These annotation errors need to be minimized because high-throughput gene calls are submitted to public databases, which in turn provide evidence for future annotations. Flatfile databases like Genebank often distinguish whether the gene calls are derived from computational annotation or laboratory experiments, meaning that findings from automated annotations could be assigned less credibility in order to reduce the amplification of erroneous calls. However, we cannot depend on hand curation and wet-lab experiments for accurate, reliable annotation as the rate of sequencing continues to accelerate. The future of genomics lies in high-throughput technology, yet no single annotation service is presently accurate enough for us to rely on exclusively. Consequently, the scientific community needs to have the ability to efficiently review the output of automated annotation services while the automation is improved.

Programs such as the Integrated Microbial Genomes Expert Review (IMG-ER) system exemplify movement in the right direction for accurate annotations. This system allows scientists to review and correct automated annotations using the IMG tools and interface. However, IMG-ER uses only one automated annotation and we are convinced that the most efficient way to substantially decrease annotation error is to compare results from multiple annotation services. Aggregating data and displaying discrepancies between annotations would present reviewers with an extensive number of possible errors including false positives, uncalled genes, genes without a predicted function, incorrectly predicted functions, and incorrect start sties. To accomplish multi-annotation comparison, information must be interchangeable between annotations. We recommend that data are accessible and sortable, and that classification is consistent. We also recommend that software be built to connect annotations in a manner that promotes easy human review. Tools that cross-query annotations and provide side-by-side comparisons will aid the user and decrease the amount of time required to make an accurate correction.

**Figure 7:**
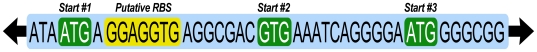
Potential comparison tool. Hypothetical comparison of start sites from multiple annotations, combined with species-specific RBS data. Start #2 would be the most likely start codon based on RBS spacing.

For example, a tool that could offer visual representation of multi-annotation comparison might clear up the difficult task of determining a gene's start site. The 5′ ends of genes are not highly conserved, and genomic databases are full of genes with incorrect start calls [22]. We might solve this problem through analysis of start codon and RBS data (Figure 7). A combination of data from multiple annotations presented visually would possibly ease the task of choosing the correct start site for a gene. Tools developed in this vein would aid the field of genomics and science as a whole.

The analysis of a genome by one of the current annotation services is not sufficient to obtain a complete analysis. Reliance on one service will likely amplify erroneous entries in genomic databases. Increased universality of data and comparison of multiple annotations are recommended in order to capitalize on the incredibly powerful opportunities offered by the field of genomics.

## Materials and Methods

We received the *H. utahensis* strain AX-2 genome sequence in FASTA format from the Joint Genome Institute. The genome had been sequenced by JGI as part of the Genomic Encyclopedia of Bacteria and Archaea (GEBA) project in conjunction with an effort to enhance undergraduate education. Whole-genome shotgun sequencing resulted in 5 contigs of varying sizes with the largest spanning 3,102,403 base pairs and representing over 99 percent of the genomic DNA. Four additional contigs measured 10,409, 9,346, 3,888, and 3,515 base pairs respectively. JGI annotated the genome through their Integrated Microbial Genome Expert Review system (IMG/ER), and made the analysis publicly available on the IMG/EDU site version 2.6 (http://imgweb.jgi-psf.org/cgi-bin/img_edu_v260/main.cgi?section=TaxonDetail&page=taxonDetail &taxon_oid = 2500575004).

We submitted the *H. utahensis* genome sequence to two additional automated annotation services: The National Microbal Pathogen Data Resource's (NMPDR) Rapid Annotation using Subsystems Technology (RAST) server (http://rast.nmpdr.org/) and the J. Craig Venter Institute (JCVI) Annotation Service (http://www.jcvi.org/cms/research/projects/annotation-service/). The RAST server provided a fully automated annotation of the genome that was viewable by the SEED-viewer [22]. The JCVI Annotation Service ran the genome through its Prokaryotic Annotation Pipeline and uploaded the output to Manatee, JCVI's web-based annotation tool and browser (http://manatee.sourceforge.net/).

### Web-based tools

We used numerous web-based tools in order to investigate the *H. utahensis* genome annotation, as well as to compare the three annotation services. We utilized features built into the IMG, RAST, and Manatee browsers, including sequence exporters, open reading frame visualizers, internal BLAST, and other search and comparison tools.

We also utilized a number of other existing web-based tools. We used the National Center for Biotechnology Information's (NCBI) BLAST tools to compare sequences across extensive databases (http://blast.ncbi.nlm.nih.gov/Blast.cgi). We visualized pathways in *H. utahensis* and related organisms by using the Kyoto Encyclopedia of Genes and Genomes (KEGG) (www.genome.jp/kegg/). We found a consensus ribosomal binding site sequence using RSAT's Pattern Discovery Tool (http://rsat.ulb.ac.be/rsat/). In order to understand discrepancies in tRNA calls, we studied tRNAscan-SE, a tool used by all three annotation services (http://lowelab.ucsc.edu/tRNAscan-SE/). We also utilized EMBOSS's “palindrome” tool to help locate the genome's origin of replication (http://emboss.bioinformatics.nl/cgi-bin/emboss/palindrome). Palindrome searches a DNA sequence to locate inverted repeats.

Additionally, we developed our own software tools to facilitate exploration of the genome and comparison of the three annotations. One tool enabled us to search all three annotations for a specific enzyme using Enzyme Commision (EC) numbers (http://www.bio.davidson.edu/courses/genomics/2008/Win/ec/). With another tool, we could BLAST multiple enzyme sequences against the genome by entering an EC number (http://gcat.davidson.edu/Wideloache/Webfiles/ecNumBlast.html). The program used enzyme information from exPASy (http://expasy.org/enzyme/) and retrieved enzyme sequences from the UniProt database (http://www.uniprot.org/). We also developed a text-based search of all three annotations' protein calls simultaneously to expedite manual searches that validated the automated gene calls (http://gcat.davidson.edu/Wideloache/Webfiles/AnnotationSearcher.html). We developed a course wiki page for all of our tools as well as to share findings, compile resources, and post questions (http://gcat.davidson.edu/GcatWiki/index.php/Halorhabdus_utahensis_ Genome). The wiki allowed us to work individually on a group project without duplicating efforts and to share new information with classmates working asynchronously.

### Wet-lab procedures

We obtained *H. utahensis* strain AX-2 (DSM 12940^T^) from Deutsche Sammlung von Mikroorganismen und Zellkulturen (http://www.dsmz.de/) and aerobically cultured the cells in 500 ml Erlenmeyer flasks containing 100 ml GSL-2 medium (NaCl, 200 g/L; citric acid, 0.5 g/L; yeast extract, 1.0 g/L; trypticase peptone, 1 g/L; 100 mL/L salt solution (10 g/L MgSO_4_ x7H_2_O; 5 g/L KCl; 2 g/L NH_4_Cl; and 1 g/L NaHCO_3_); 2 ml/L trace metal solution TMS 3 [24] (2 ml/L FeCl_2_/MnCl_2_ solution; 2 ml/L CaCl_2_ solution; 10 ml/L KH_2_PO_4_ solution; 884 ml/L Milli-Q water, and 3 g/L of a macromolecular substrate (Soluble starch, ACS reagent (Sigma, S-9765); Birchwood xylan, (Carl Roth, Germany); CMC-Na (Sigma, C-9481); Avicel, PH-101 (Fluka, 11363); Alpha-cellulose (Sigma, C-8002); or crab-shell chitin (Sigma, C-9752)). Control flasks contained only GSL-2 medium devoid of macromolecular substrate. The pH of the complete medium was adjusted to 6.9 using NaOH. The medium was sterilized by autoclaving at 121°C for 25 minutes. We inoculated the culture flasks with *H. utahensis* preculture (5% v/v), and incubated for 7 days at 40°C and 120 rpm. To test whether a temperture shift would induce different results, the cells were incubated an additional 3 days at 50°C and 120 rpm. We evaluated growth by noting the presence of *H. utahensis*' red coloring and checking for cells by microscopy.

Additionally, we tested the culture flasks for chitinase, mannanase, xylanase, cellulase, pullulanase, and amylase activity using enzyme assays. We carried out enzyme assays as described by Wainø and Ingvorsen (2003) [25], using Chitin Azure (Sigma, C-3020), Colloidal-Chitin-RBV, AZCL-Galacto-mannan (Carob), AZCL-Arabino-xylan, AZCL-Hydroxyethyl-Cellulose, Ostazin-Brilliant Red-Hydroxyethyl-Cellulose (Sigma, O-6879), AZCL-Xylan (Birchwood), AZCL-Pullulan, and AZCL-Amylose. We obtained all insoluble polymer AZCL-substrates from Megazyme (Ireland).
